# EDUCATIONAL GRADIENTS IN HEALTH AND COGNITION: DOES THE TIMING OF EDUCATIONAL ATTAINMENT MATTER?

**DOI:** 10.1093/geroni/igae098.2661

**Published:** 2024-12-31

**Authors:** Fabio Bolz, John Warren, Eric Grodsky, Chandra Muller

**Affiliations:** University of Minnesota, Minneapolis, Minnesota, United States; University of Minnesota, Minneapolis, Minnesota, United States; University of Wisconsin Madison, Madison, Wisconsin, United States; The University of Texas Austin, Austin, Texas, United States

## Abstract

Educational attainment is a strong predictor of later-life health and cognition. However, studies on the relationship between educational attainment and these outcomes usually do not consider the role of the timing of educational attainment. There is considerable heterogeneity in the timing of college degree attainment in the US and there are pronounced differences in timing by gender and race. Using nationally representative longitudinal data from High School and Beyond (HSB) we ask: (1) Does the impact of educational attainment on midlife health and cognition vary by age of degree attainment? (2) Does age at degree attainment moderate the effects of education differently across racial/ethnic and gender groups? We estimate OLS and logistic regression models for a variety of health and cognitive outcomes including: hypertension, diabetes, mental health conditions, general cognition, immediate recall, semantic fluency, phonemic fluency, delayed recall and working memory. We control for an extensive set of potentially confounding variables such as cognitive skills and non-cognitive traits in adolescence, academic performance in high school, and health in adolescence. Preliminary results suggest that bachelor’s degrees obtained later in life are associated with substantially lower levels of cognitive functioning at midlife than degrees obtained earlier in life; however, people who obtain college degrees after age 40 still display higher levels of cognitive functioning than individuals without a bachelor’s degree. Rates of hypertension and diabetes among those who obtained their college degree after age 30, in contrast, do not differ from rates for individuals without college degrees.

